# Decentering the Self? Reduced Bias in Self- vs. Other-Related Processing in Long-Term Practitioners of Loving-Kindness Meditation

**DOI:** 10.3389/fpsyg.2016.01785

**Published:** 2016-11-21

**Authors:** Fynn-Mathis Trautwein, José R. Naranjo, Stefan Schmidt

**Affiliations:** ^1^Department of Social Neuroscience, Max Planck Institute for Human Cognitive and Brain SciencesLeipzig, Germany; ^2^Department of Psychosomatic Medicine and Psychotherapy, Medical Faculty, University Medical Center FreiburgFreiburg, Germany; ^3^Brain Products GmbHMunich, Germany; ^4^Institute of Transcultural Health Studies, European University ViadrinaFrankfurt (Oder,) Germany

**Keywords:** meditation, loving-kindness, compassion, self, P300

## Abstract

Research in social neuroscience provides increasing evidence that self and other are interconnected, both on a conceptual and on an affective representational level. Moreover, the ability to recognize the other as “like the self” is thought to be essential for social phenomena like empathy and compassion. Meditation practices such as loving-kindness meditation (LKM) have been found to enhance these capacities. Therefore, we investigated whether LKM is associated to an increased integration of self–other-representations. As an indicator, we assessed the P300 event-related potential elicited by oddball stimuli of the self-face and a close other’s face in 12 long-term practitioners of LKM and 12 matched controls. In line with previous studies, the self elicited larger P300 amplitudes than close other. This effect was reduced in the meditation sample at parietal but not frontal midline sites. Within this group, smaller differences between self- and other-related P300 were associated with increasing meditation practice. Across groups, smaller P300 differences correlated with self-reported compassion. In meditators, we also investigated the effect of a short LKM compared to a control priming procedure in order to test whether the state induction would additionally modulate self- vs. other-related P300. However, no effect of the priming conditions was observed. Overall, our findings provide preliminary evidence that prolonged meditation practice may modulate self- vs. other-related processing, accompanied by an increase in compassion. Further evidence is needed, however, to show if this is a direct outcome of loving-kindness meditation.

## Introduction

When René Descartes declared “cogito ergo sum,” he referred to the inherent relation of mental phenomena to the solipsistic thinking self. This idea is still reflected in a wealth of modern psychology and neuroscience research, which characterizes mental and neuronal processes as being centered on a core structure referred to as “the self.” Within this paradigm it has been shown that attention, memory, and motivation are biased by the degree to which information is related to or relevant for the self (Wood and Cowan, 1995; Symons and Johnson, 1997; Crocker and Park, 2003; Sui and Humphreys, 2015). However, contemplative scholars and some philosophers have emphasized that “the other” is inherently connected with the self (Husserl, 1960; Buber, 1995; Wallace, 2001). More recently, social neuroscience has pursued this view, suggesting that an overlap of self and other representations underlies human intersubjectivity (Decety and Sommerville, 2003; Gallese, 2003). Increasing evidence suggests that mental training through meditation fosters intersubjective skills (Mascaro et al., 2015). Specifically, practices such as loving-kindness meditation (LKM) may increase social connectedness (Hutcherson et al., 2008), empathy (Mascaro et al., 2013), compassion (Klimecki et al., 2013, 2014), emotional resonance (Lutz et al., 2008), positive affect (Fredrickson et al., 2008), and altruism (Leiberg et al., 2011; Condon et al., 2013; Weng et al., 2013). However, little is known about the underlying mechanisms. Hence, we investigated whether the practice of LKM involves a rebalance of self- and other-related processing.

Several lines of research indicate that self and other are co-represented in “shared” neural networks, including the mirror-neuron system, emotion circuits, and cortical midline structures (Uddin et al., 2007; Bernhardt and Singer, 2012). While the former two networks support representation of bodily and affective states, cortical midline structures are related to conceptual reflection on self and other. Various factors seem to influence the degree to which these processes, mostly recruited by the self, are also recruited by the other. For example, cultures which promote an interdependent, socially embedded conception of the self extend the focus on an individual self commonly observed within Western cultures toward socially relevant others, thereby diminishing differences in neural signatures related to self and other (Han and Northoff, 2008). Furthermore, also Westerners integrate close others into the self (Aron et al., 2004) and differences exist in the degree to which individuals tend to define themselves in terms of their interpersonal relationships (Cross et al., 2002). A meta-analysis of neuroimaging studies involving trait evaluation paradigms corroborates this view. Across 25 studies, both self-reflection and close-other-reflection recruited the medial prefrontal cortex, whereas this was not the case for reflection upon familiar, but not personally known others (Murray et al., 2012). Furthermore, some evidence shows that mindfulness meditation, which is often practiced in conjunction with LKM, attenuates conceptual self-referential activity (Farb et al., 2007; Berkovich-Ohana et al., 2012; Dor-Ziderman et al., 2013). This technique involves focusing on current somatosensory and mental events in a non-conceptual, non-judgmental manner, and is thought to cause a detachment from a solid and independent sense of identity (Hölzel et al., 2011).

Another line of research indicates that the degree of self–other overlap on a conceptual level is related to the extent of resonance in shared affective networks, such as when confronted with the suffering of others (Hein et al., 2010; Meyer et al., 2012). Reflecting this close link between affective and conceptual levels of self–other integration, a recent study (Liu et al., 2013) found that administration of oxytocin—a hormone related to prosocial affect and behavior—induced a rebalance of self- and other-related processing. Specifically, the study found a relative reduction of later positive event-related potential (ERP) amplitudes (220–280 and 520–1000 ms) during self-related trait judgments but an increase during other-related judgments. Similarly, a rebalance of self- vs. other-related processing might also be involved in the cultivation of prosocial affect through meditation as done in LKM. Such an effect could serve as an underlying mechanism for previously reported outcomes in the social domain, including social connectedness (Hutcherson et al., 2008), empathy (Mascaro et al., 2013), compassion (Klimecki et al., 2013, 2014), and altruism (Leiberg et al., 2011; Condon et al., 2013; Weng et al., 2013). Furthermore, as pointed out below, the phenomenology of LKM is also consistent with such a view.

Thus, meditation might modulate self-related processes in a twofold way (cf. Trautwein et al., 2014): (1) attenuation of self-referential activity on a conceptual level (mindfulness meditation); (2) enhancement of self–other integration via an affective route (LKM). While contemplative accounts emphasize interrelatedness of both of these processes (Salzberg, 2011), the latter was the focus of the present study.

Specifically, here we focus on the practice of LKM (see Section “Materials and Methods” for a definition in the context of our study). The word “meditation” in general designates a group of practices for the self-regulation of body and mind (Schmidt, 2014). Meditation supposedly induces transient state effects during and directly after the practice itself as well as more persistent trait changes through regular practice (Lutz et al., 2007; Tang et al., 2015). LKM originated in Buddhism and aims at the cultivation of “metta,” an unconditional and impartial kindness toward the self and others (Buddharakkhita, 1995; Salzberg, 1995). The practice involves generating a heartfelt wish for the well-being of oneself and others by means of inner verbalizations (e.g., “may you be happy”) or visualizations (e.g., imagining a close person). Typically, practitioners begin with themselves or a close other and then extend the wishes toward a widening circle of others, including unknown and “difficult” persons. Thus, this practice involves relating to the self as “like the other” as well as seeing the other as “like the self” (Wallace, 2001). Often LKM is practiced together with similar techniques such as compassion meditation, i.e., cultivating the wish to relieve other’s suffering (Salzberg, 1995). While there is an increasing number of empirical studies regarding the effects of LKM, so far it has not been tested whether a stronger self–other integration indicated by a rebalance of self- and other-related processing is involved in these practices. Some indirect evidence comes from a study of a Buddhist sample, which, however, did not require participants to be engaged in regular meditation practice (Colzato et al., 2012). As an indicator of self–other integration, the study investigated the social Simon effect, which indexes interference caused by automatically co-representing another individual’s actions (i.e., self–other overlap in the motor domain). While the study found a significant difference between Buddhists and a matched control sample, it is unclear how this effect is related to the practice of meditation and whether it generalizes to other domains of self–other integration.

To assess self–other integration, we chose a different measure based on P300 ERPs elicited by pictures of the self and of a close other. The P300 is a broadly distributed positivity in the electroencephalogram (EEG) with a centroparietal maximum occurring 300–600 ms after stimulus presentation (Comerchero and Polich, 1999). The P300 is observed after infrequent target stimuli and salient distracters, while being absent after frequent standard stimuli; and it is thought to reflect domain general post-perceptual processes. Similar to other attentional and cognitive biases in self-related processing (e.g., Sui et al., 2013; Humphreys and Sui, 2015), previous studies consistently found larger P300 amplitudes for self-related stimuli including one’s own name, face, autobiographical information, and self-related pronouns compared to not self-related control stimuli (Gray et al., 2004; Perrin et al., 2005; Zhao et al., 2009, 2011; Tacikowski and Nowicka, 2010; Zhou et al., 2010; Fan et al., 2013). Similar P300 effects of autobiographical stimuli have been shown to occur highly automatic and have thus been used for detection of concealed memories (Labkovsky and Rosenfeld, 2012; Meixner and Rosenfeld, 2014). The rationale for choosing this measure as an indictor of self–other integration was the following: if the self representation of an individual is less isolated but instead embedded more into a social context, then self- and other-related stimuli should also elicit more similar cognitive processing. The P300 elicited by self- and other faces thus seemed an ideal global and implicit marker of self–other integration.

As a self-related stimulus, pictures of participants’ own faces were chosen because the self-face is often used to investigate neural correlates of the self (Devue and Brédart, 2011). Moreover, neuronal processing of self vs. other faces has been found to be modulated by the kind of culturally shaped self-construal, which varies between independent, individualistic and interdependent, socially integrated styles (Sui and Han, 2007; Sui et al., 2009), the latter being related to our conception of self–other integration (cf. Dambrun and Ricard, 2011). Furthermore, face processing involves both bodily and conceptual processes (Uddin et al., 2007), and will thus potentially capture self–other overlap on both levels. Assuming that cultivation of prosocial mental qualities through LKM shifts the focus of mental processes from the individual self to increasingly integrate self and other, we hypothesized that reduced differences between self- and other-related P300 amplitudes should be associated to this practice.

**FIGURE 1:**
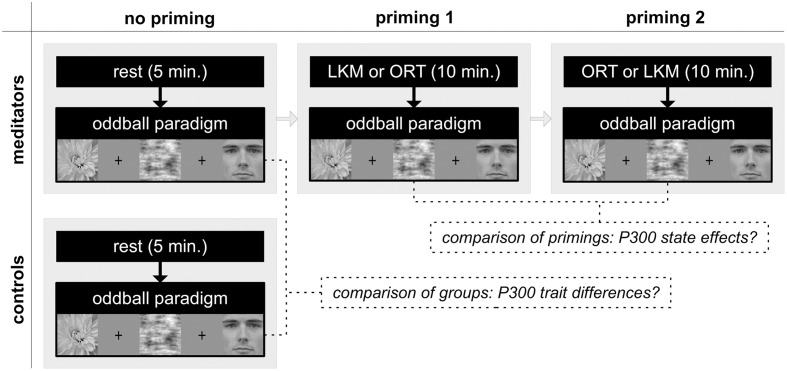
**The design of the present study.** LKM, loving-kindness meditation; ORT, other-referential thinking. See text for further explanations.

The current study assessed this hypothesis in a twofold way (see **Figure 1** for the design of the study): (1) trait changes due to continuous practice of LKM were assessed by comparing 12 long-term practitioners of LKM to a closely matched control group prior to any meditative state. (2) To assess state effects of LKM, long-term meditators additionally went through two priming conditions in a counterbalanced order, a short LKM and a control state (other-referential thinking, ORT, see Materials and Methods). ERPs were recorded directly after these primings. Priming effects were investigated only in meditators based on the assumption that state effects would be stronger and thus, changes in self- and other-related processing would be easier detectable in trained practitioners of LKM.

With respect to self-report measures we hypothesized that meditators would experience more compassion for the self and for others in everyday life than controls (hypothesis 1). These traits were assessed by the Self-Compassion Scale (SCS; Neff, 2003a) and the Compassionate Love Scale (CLS; Sprecher and Fehr, 2005). Regarding ERP data we hypothesized that, compared to controls, meditators would show reduced differences between self- and other-related P300 (hypothesis 2). In meditators, these differences were expected to be smaller after a short LKM session in the lab as compared to a control procedure (hypothesis 3). Furthermore, we predicted that differences between self- and other-related P300 would be correlated negatively with questionnaire scores of compassionate love for the self and others (hypothesis 4). This follows from the notion that an overlap of self and other representations is involved in the experience of empathy and compassion (Preston and Hofelich, 2012), but also from psychological descriptions of these concepts. For example, according to Neff (2008), self-compassion entails a de-emphasis of the individual self in favor of shared aspects of identity. Finally, within meditation practitioners we expected that higher amounts of meditative practice would be associated with smaller differences between self- and other-related P300 (hypothesis 5).

## Materials and Methods

### Participants

Twenty-four healthy volunteers participated in this study, 12 long-term meditators and 12 matched controls (see **Figure 1** for the design). Following ERP assessment without any priming, only meditators also completed assessments after each of two priming tasks. All participants had normal or corrected-to-normal vision. Meditators were recruited from local meditation centers and all of the following inclusion criteria were required: (a) general meditation practice on a regular basis (at least once a week) during the last 2 years or more; (b) LKM practice on a regular basis (at least once a week) during the last 3 months or more; (c) LKM practice includes explicit engagement in developing loving-kindness for oneself and for specific others. The control group was recruited after the meditation group and matched for age, sex, handedness, and education. Inclusion criterion was to have no prior meditation experience. The study was approved by the ethics committee of the University Medical Center Freiburg and carried out in compliance with the Declaration of Helsinki. Participants gave written informed consents and were paid commensurate with the amount of time invested for participation (30 € meditators; 20 € controls).

### Priming Conditions

Prior to two additional ERP assessments, meditators completed two priming conditions. The order of the two primings was counterbalanced across subjects. The instruction for the LKM priming was to generate loving-kindness toward oneself and, after approximately 2.5 min, to direct loving-kindness toward a specific close other for the remaining time of a 10-min period. For the control condition (ORT), instructions were to think for 2.5 min in an emotionally neutral manner about oneself and then about the close other (e.g., about personal characteristics). Participants were asked to keep eyes closed during these tasks.

### Stimulus Preparation

Prior to the experiment, participants handed in one photograph of their own and one of a close other’s face. The close other was defined as a person to whom the participant has a close and positive relationship, e.g., a good friend, is of the same sex as the participant, and is not a family member. Meditators were asked to choose a person who would facilitate the development of loving-kindness when taken as the subject of LKM. The photographs had to show a frontal view of the face with no emotional or other facial expression and no object covering parts of the face. The photos were scaled to a standard mask, which defined the pupil to mouth distance and the midline of the face (see **Supplementary Figure S1** for mask and example stimuli). Images were converted to gray scale and cropped to 240 by 240 pixels. To ensure similar brightness across stimuli, mean luminosity was anchored to a fixed level. A picture of a flower was equally processed as the face pictures. Scrambled images were created from the self face, the other face, and the flower image by use of an algorithm, which synthesizes a texture from an original image by randomizing its Fourier phase (Galerne et al., 2011).

### Oddball Paradigm

E-Prime^®^ (version 2; Psychology Software Tools, Inc.) was used to present stimuli in central vision on a 17-in. LCD monitor at 100 cm distance (screen resolution: 800 × 600; stimulus size: 10.2 × 10.8 cm; visual angle: 5.8° × 6.2°) on a gray background. Within the three-stimulus oddball paradigm (e.g., Jeon and Polich, 2001), the picture of the participant’s own face and the picture of the close other’s face served as distracters. During the LKM priming, participants were asked to direct the feeling of loving-kindness toward the same person as depicted on the picture. A picture of a flower was the target stimulus requiring a button press. Scrambled versions of these stimuli served as standards. In order to minimize local sequence probabilities of target and distracter stimuli (Polich and Bondurant, 1997), a pseudo-randomized stimulus sequence was created for each session with the following stimulus frequencies and constraints: 20% target, 60% standards (20% each), 20% distracters (10% each); no immediate succession of distracter stimuli; maximal two successive target stimuli; maximal six successive standard stimuli.

Recording sessions consisted of two blocks of 375 stimuli, resulting in a total of 750 stimuli. Stimuli were presented for 100 ms followed by a fixation cross for a duration that varied randomly between 1200, 1500, or 1800 ms. Participants were instructed to keep their eyes focused on the fixation cross and to press a button with the index finger of the dominant hand when the target stimulus appeared. Response speed was emphasized but not at the cost of accuracy.

### Procedure

The study took place in the neurophysiological laboratory of the Research Center for Meditation Mindfulness and Neurophysiology at the University Medical Center Freiburg, Germany. At arrival, participants gave informed consent and filled out questionnaires. Thereafter, participants were accompanied to the EEG lab and seated in an electrically and acoustically shielded chamber. After electrode application and impedance reduction, instructions were presented on the monitor, followed by a short training block of 20 stimuli (same stimuli as during the experiment). First, rest EEG was measured for 5 min with eyes closed and instructions to relax without purposefully engaging in any particular mental activity. For both groups, two stimulus blocks lasting 10 min each were then presented with a short pause of 1 min in between. Thereafter, only meditators remained in the lab, read instructions for the first priming and engaged in the specified task (LKM or ORT, applied in a counterbalanced order). Subsequently, two stimulus blocks were presented, followed by the second priming task and another recording session. At the end, participants filled out a short questionnaire about the measurement and were dismissed.

### Self-Report Measures

To assess self-reported compassion for others in general and for the close other shown on the picture, we applied specific other and stranger-humanity versions of the CLS (Sprecher and Fehr, 2005; German *ad hoc* translations also applied by Leiberg et al., 2011). To assess closeness between participants and the person shown on the picture, the Inclusion of Other in the Self Scale (IOS; Aron et al., 1992) was used. Self-compassion, the ability to relate to the self in a caring way by recognizing shared aspects of identity (Neff, 2008), was assessed using the SCS (Neff, 2003b; German translation by Bartel, 2009; validated by Spottke, 2013). Scale ranges for the CLS and IOS were 1–7 and 1–5 for the SCS.

*Ad hoc* questionnaires were used for sociodemographic characteristics and meditation experience. Additionally, the following potentially confounding variables concerning self- and other-related ERPs were assessed: years of acquaintance with close other, frequency of meeting, recency of last meeting, similarity [visual analog scale (VAS) with anchors “very dissimilar” vs. “very similar”], pleasantness of self and other images (VAS with anchors “very negative” vs. “very positive”; assessed after EEG recordings).

### EEG Data Recording

Continuous EEG was recorded from 64 scalp sites using the actiCap electrode system, a 72-channel amplifier (QuickAmp) and BrainVisionRecorder^®^ software (all from Brain Products, Munich, Germany). Electrodes were positioned according to the extended International 10–20 System. The EEG signal was recorded against an average of all channels calculated by the amplifier hardware, with the ground placed at the chin. For electrooculogram (EOG) monitoring, two bipolar electrodes were placed at the outer canthi of both eyes and another two above and below the left eye. Impedance was always kept below 10 kΩ and mostly below 5 kΩ. Signals were recorded with a sampling rate of 1000 Hz and a low-pass filter with cut-off at 280 Hz.

### EEG Data Analysis

EEG analysis was done in BrainVision Analyzer^®^ 2.0 (Brain Products, Munich, Germany) by keeping to the recommendations of Duncan et al. (2009). A zero-phase Butterworth high-pass filter (24 dB/octave) at 0.01 Hz was applied. EEG data was re-referenced to linked mastoid electrodes (TP9, TP10) and segmented into 1000 ms epochs (200 ms pre-stimulus to 800 ms post-stimulus). Data was aligned to the 200 ms pre-stimulus baseline and corrected for ocular artifacts (Gratton et al., 1983). For artifact reduction, all trials exceeding ±100 μV were rejected. We exported mean P300 amplitudes of five midline electrodes (Fz, FCz, Cz, CPz, and Pz) for statistical analysis into SPSS^®^ (version 20). In order to avoid biases for specific group or stimulus conditions, the mean amplitude window was defined by assessing the period of maximal global field power of the P300 component across participants and conditions (Lehmann and Skrandies, 1984; Picton et al., 2000). Furthermore, latencies were measured as local peaks of the global field power between 300 and 600 ms.

### Statistical Analysis

#### ERP data

For group effects, P300 amplitudes were analyzed in a three-way analysis of variance (ANOVA) for *group* (controls vs. meditators), *stimulus* (self vs. other), and *electrode* (Fz, FCz, Cz, CPz, and Pz). State effects of LKM and ORT were tested in a three-way repeated measure ANOVA for *priming* (LKM vs. ORT), *stimulus* (self vs. other) and *electrode* (Fz, FCz, Cz, CPz, and Pz). When appropriate, degrees of freedom were adjusted according to the Greenhouse–Geisser epsilon. Significant interactions of the stimulus factor were followed up by simple (interaction) effects ANOVA (Howell and Lacroix, 2012). P300 global-field power latencies were analyzed in a two-way ANOVA for *group* (controls vs. meditators) and *stimulus* (self vs. other).

#### Self-Report Data

For group comparisons of questionnaire scores, we employed *t*-tests for independent samples. Non-parametric correlations (Spearman’s rho) were used to analyze relationships between self-report data and P300 amplitude. Furthermore, we investigated relationships between extent of individual meditation practice and P300 amplitude. Since meditation experience increases with age and thus might be confounded with developmental factors, we computed partial correlations controlling for age. Correlations are classified according to Cohen (1992) as small (0.10 ≤*r* ≤ 0.29), medium (0.30 ≤*r* ≤ 0.49), and large (*r* ≥ 0.50). Throughout the paper, effects are reported as significant at *p* ≤ 0.05. Partial eta squared values (η^2^) and Cohen’s *d* are reported as effect sizes for ANOVA and *t*-test results, respectively.

#### Behavioral Data

Note that the used experimental task was not optimized for behavioral assessment of self- and other-related processing, as responses were only required for the target (i.e., the flower picture) and not for self and other pictures. Therefore, we did not have any strong hypotheses for the behavioral data. Nevertheless, the saliency of the distracter stimuli, and especially of the self-face, might trigger responses (“false alarms”) in some trials and thereby induce a behavioral self-bias. We thus calculated the difference of false alarm rates between self and other conditions and tested for differences between groups using the Wilcoxon rank sum test as well as for differences in the within subject comparison of priming conditions using the signed rank test. Non-parametric tests were used because of the highly skewed, zero-inflated frequency data (see **Supplementary Figure S4**). Moreover, we also analyzed target hit rate and reaction time to check whether task performance differed between the two groups (e.g., due to different attentional performance).

## Results

### Sample Description

Because of extensive artifacts in the EEG recordings, one participant in the meditation group had to be excluded from the analysis. To keep both groups equal, we also excluded the matched participant from the control group from all analyses. The remaining participants (*n* = 22) were aged between 26 and 61 years (mean = 42, SD = 11). Both groups were matched for age (meditators: mean = 42 years, SD = 11, controls: mean = 41 years, SD = 10), sex (six females and five males each), handedness (10 right-handed and one left-handed each), and education. Meditators (*n* = 11) located themselves within different traditions [Theravada Buddhism (*n* = 3), Tibetan Buddhism (*n* = 6), no specific tradition (*n* = 2)]. On average, they had practiced meditation for 139 months (SD = 109, min = 24, max = 372) and LKM in particular for 106 months (SD = 98, min = 8, max = 372). Mean estimated hours of sitting meditation was 1233 (SD = 1979, min = 308, max = 7056) for LKM and 2435 (SD = 2557, min = 64, max = 7056) for meditation in general. For the last 8 weeks before the experiment, the average amount of LKM practice was 2.5 h/week (SD = 1.125, min = 0.75, max = 10.5).

### Self-Report Data

Self-report data was assessed in order to evaluate whether meditators would report to experience more compassion (hypothesis 1), as well as to assure equivalence across the two groups for the relationships between participants and the respective close other used in the oddball paradigm. **Table 1** presents an overview of results for self-report measures. In the CLS, meditators reported to experience more compassionate love for strangers and all of humanity than control participants. In contrast, compassionate love for the specific close other did not differ between groups. The SCS (*n* = 21 because of missing data for one participant) indicated higher self-compassion in meditators. Closeness as measured by the IOS did not differ between groups. These results are in line with hypothesis 1, indicating that practitioners of LKM experience more compassion for the self and for others in everyday life than controls.

**Table 1:** Descriptive and inferential statistics for self-report data.

	Meditators		Controls		*t*-test for group differences			
		
	Mean	SD	Mean	SD	*df*	*t*	*p*	*d*
CLS-H	5.00	0.65	4.08	0.86	20	2.84	**0.010**	1.21
CLS-O	5.32	0.37	5.42	0.62	20	-0.47	0.639	-0.20
SCS^a^	3.70	0.63	3.09	0.62	19	2.33	**0.030**	0.97
IOS	3.91	1.81	4.18	1.33	20	-0.40	0.692	-0.17


*CLS-H, Compassionate Love scale, stranger-humanity version; CLS-O, Compassionate Love Scale, specific close other version; SCS, Self-Compassion Scale; IOS, Inclusion of Other in Self Scale; *p*-values (two-tailed) ≤ 0.05 are highlighted in bold face type. ^a^One missing data set in meditators (*n* = 10).*

### Behavioral Data

Behavioral data was analyzed to assure similar task performance across groups and as a potential additional marker of changes in self–other related processing. For one of the control participants, no behavioral data were recorded, and hence we also excluded the matched participant in the meditation group. Furthermore, the hit rate was extremely low for one meditator during one of the priming runs (49% as compared to a median of 99% for the remaining subjects in the priming measurements), and therefore the participant was excluded in the within subject analysis of priming effects.

First, the difference between response rates (“false alarms”) for self- vs. other-face was calculated for each participant (see **Supplementary Figure S4**). These difference scores were then compared between meditators and controls as a potential additional marker of differences in self- vs. other-related processing. While, descriptively, controls showed a small self-bias (median = 1.3, range = -1.33 to 7.89) and meditators did not (median = 0, range = -1.35 to 2.63), the group difference was not significant (*U* = 71.5, *p* = 0.107). In contrast, the self minus other difference scores differed between the priming conditions (*W* = 21, *p* = 0.036), with a stronger self-bias after ORT (median = 0.66) as compared to LKM (median = 0, range = -2.70 to 1.33).

Moreover, we tested for group differences in accuracy of target detection (hit rate) and reaction time (see **Supplementary Figure S4**), in order to check for potential differences in general task performance (e.g., due to attentional differences). Yet, group differences existed neither in accuracy (*U* = 55.5, *p* = 0.686) nor in reaction time (*t* = 0.64, df = 18, *p* = 0.528).

### Event-Related Potentials

#### Group Comparison

**FIGURE 2:**
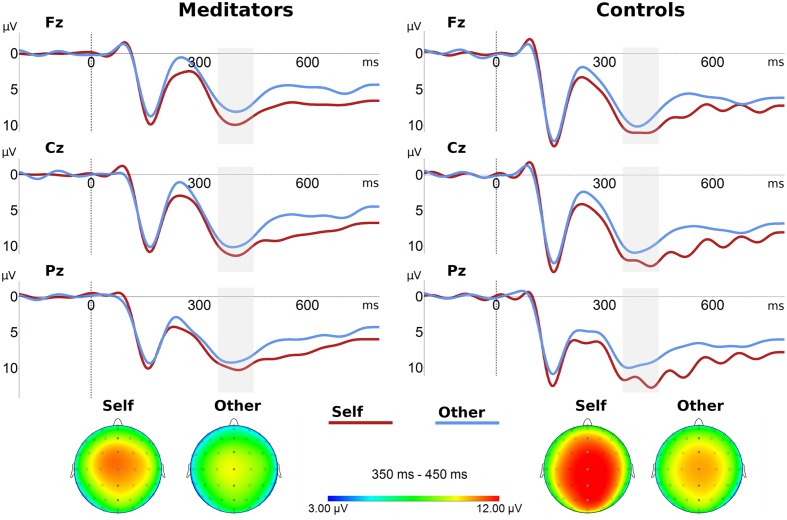
**Grand average ERPs and topographic distributions of P300 components for self stimulus and other stimulus.** *Top*: ERPs elicited at midline electrodes (Fz, Cz, and Pz). Stimulus onset occurred at 0 ms. *Bottom*: Topographic distributions of P300 mean amplitudes (350–450 ms, gray area in ERP plots).

To test for trait-like increases in self–other integration in meditators, we compared this group with controls prior to any meditative state or priming, expecting reduced differences between self- and other-related P300 in meditators (hypothesis 2). As depicted in **Figure 2**, P300 components centered on centroparietal areas emerged for both distracter stimuli. The global field power of distracters indicated a maximum of this component from 350 to 450 ms (**Supplementary Figure S2**), which was chosen for mean amplitude assessment.

**FIGURE 3:**
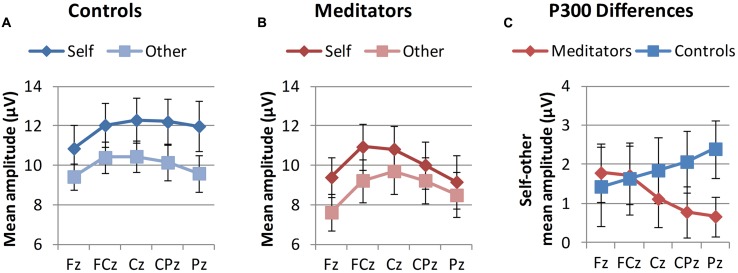
**Group means of P300 mean amplitudes at midline electrodes.** **(A,B)** Show P300 mean amplitudes for self and other stimuli in both groups. **(C)** Shows the group averages of P300 mean amplitude differences for self minus other. Error bars represent standard error of the mean.

Regarding P300 mean amplitudes (**Figure 3**; **Table 2**), a three-way ANOVA [*group* (controls vs. meditators) × *stimulus* (self image vs. other image) × *electrode* (Fz, FCz, Cz, CPz, and Pz)] yielded the following results: the main effect of *group* was not significant, indicating that P300 amplitudes for the distracter stimuli were, overall, equal for controls and meditators. A significant main effect of *electrode* appeared, reflecting the focal maximum of the component at Cz. Regarding the main effect of *stimulus*, larger P300 amplitudes for self image vs. other image were observed, replicating prior studies of effects of self-related stimuli on P300 (e.g., Gray et al., 2004). The interaction *group* × *stimulus* was not significant, indicating that averaged across electrodes groups did not differ in self- vs. other-related processing. However, a significant three-way interaction of *stimulus* × *electrode* × *group* indicated spatially dependent differences in self vs. other-related processing between both groups. Decomposing these effects into simple interactions of group by stimulus showed the strongest effect at Pz (*F*_1,20_ = 3.60, *p* = 0.072, critical significance). Simple main effect analysis demonstrated that, in controls, differences between self and other were highly significant at Pz (*F*_1,20_ = 13.85, *p* = 0.001). In meditators, however, these differences were not significant (*F*_1,20_ = 1.08, *p* = 0.31). In order to investigate the robustness of these results, we performed an analysis with pooled values from electrodes centered around the region where the *group* × *stimulus* interaction was maximal (Pz, POz, P1, P2, PO3, PO4). Again, a close to significant *group* × *stimulus* interaction was observed (*F*_1,20_ = 3.42, *p* = 0.079), pointing toward differences between self and other in controls (*F*_1,20_ = 12.92, *p* = 0.002), and no significant differences in meditators (*F*_1,20_ = 0.96, *p* = 0.40). Qualitatively, topographies and interaction diagrams (**Figures 2** and **3**) suggest that the three-way interaction was mainly characterized by a frontocentral peak for the self-face in meditators in contrast to more dispersed central to centroparietal focus in controls. We therefore additionally tested for the *group* × *electrode* interaction separately in the self-face condition, which yielded a critically significant effect (*F*_1.32,26,43_ = 1.18, *p* = 0.056); which was not the case in the other-face condition (*F*_1.61,32.21_ = 0.44, *p* = 0.606).

**Table 2:** ANOVA results for P300 mean amplitudes.

Factor	*df*	*F*	*p*	η^2^
**Group effects**				
Group	1, 20	1.26	0.276	0.06
Stimulus	1, 20	8.39	**0.009**	0.30
Electrode	1.41, 28.29	4.24	**0.036**	0.18
G × S	1, 20	0.54	0.542	0.02
G × E	1.41, 28.29	0.36	0.631	0.02
S × E	2.30, 46.01	0.40	0.702	0.02
G × S × E	2.30, 46.01	7.70	**0.001**	0.29
**Priming effects**				
Priming	1, 10	0.65	0.439	0.06
Stimulus	1, 10	3.38	0.096	0.25
Electrode	1.33, 13.30	4.16	0.053	0.29
P × S	1, 10	0.40	0.544	0.04
P × E	1.66, 16.64	0.42	0.630	0.04
S × E	2.20, 22.02	0.54	0.609	0.05
P × S × E	2.19, 21.87	1.02	0.384	0.09


**p*-values ≤ 0.05 are highlighted in bold face type.*

P300 peak latencies were significantly shorter for *other* (413 ms) compared to *self* (438 ms) stimuli (*F*_1,20_ = 5.94, *p* = 0.024). No significant *group* effect (*F*_1,20_ = 0.18, *p* = 0.70) or *group* × *stimulus* interaction (*F*_1,20_ = 0.74, *p* = 0.399) was present.

Group effects on P300 amplitudes do not fully support the hypothesis that differences between self- and other-related P300 are in general smaller in LKM practitioners (hypothesis 2). However, they provide evidence that spatially dependent differences between groups exist in self- vs. other-related processing, with a smaller self bias in meditators toward parietal areas.

In order to test for group effects which are not specific to the self-relatedness of the stimulus, but may depend on the stimulus category (target, distracter, standard), we also analyzed ERPs elicited by these stimuli. Targets elicited a P300 component that showed a maximum of global field power between 400 and 500 ms (see **Supplementary Figures S2** and **S3**), while standards did not elicit P300 components. For statistical analysis (see **Supplementary Table S1**), within category differentiations were dropped as they are analyzed in the main analysis (self vs. other) or did not show differential effects (scrambled stimuli). Most importantly, the main effect of *group* as well as *group* × *stimulus* × *electrode* interactions were not significant, which supports the conclusion that groups differed mainly in respect to self- and other-related processing.

#### Priming Effects

**FIGURE 4:**
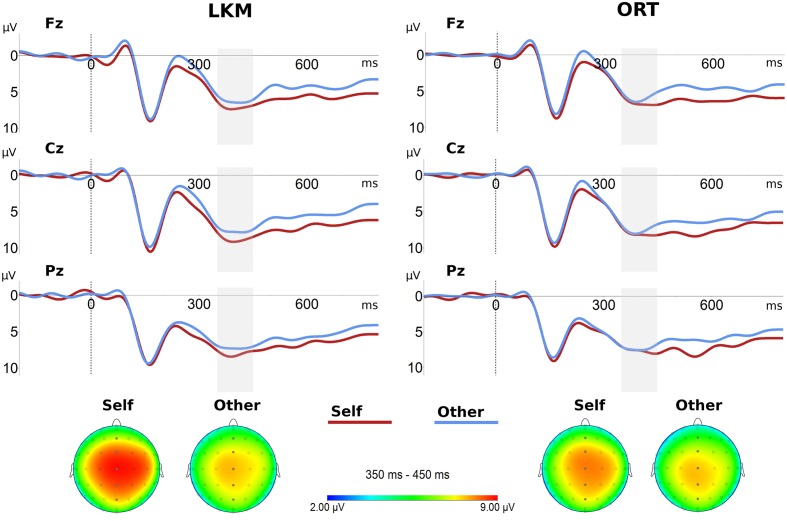
**Grand average ERPs and topographic distributions of P300 components for self stimulus and other stimulus after priming conditions.** *Top*: ERPs elicited at midline electrodes (Fz, Cz, and Pz) after loving-kindness meditation (LKM) and other-referential thinking (ORT). *Bottom*: Topographic distributions of P300 mean amplitudes (350–450 ms, gray area in ERP plots).

**FIGURE 5:**
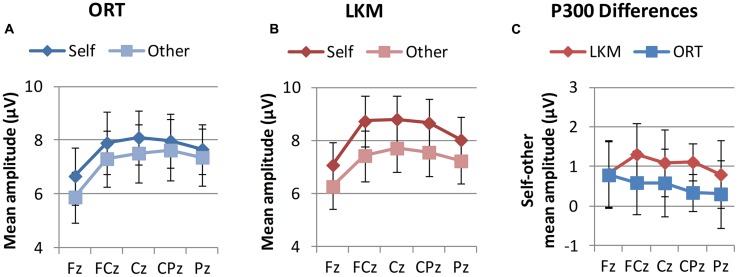
**Group means of P300 mean amplitude at midline electrodes after priming conditions.** **(A,B)** Show P300 mean amplitudes for self and other stimuli in the two priming conditions. **(C)** Shows the priming condition averages of P300 mean amplitude differences for self minus other. LKM, loving-kindness meditation; ORT, other-referential thinking. Error bars represent standard error of the mean.

ERPs recorded after the primings (ORT or LKM) were assessed to evaluate whether LKM would increase self–other integration as indicated by reduced differences between self- and other-related P300 (hypothesis 3). Descriptively, ERPs showed typical P300 components (**Figure 4**). A three-way ANOVA [*priming* (ORT vs. LKM), *stimulus* (self vs. other), *electrode* (Fz, FCz, Cz, CPz, and Pz)] of mean amplitudes (**Figure 5**; **Table 2**) yielded no significant main effects for *priming* and *stimulus*, though there was a trend toward higher amplitudes for the self stimulus. A close to significant main effect of *electrode* (*p* = 0.053) appeared, reflecting the central maximum of the component. None of the two-way or three-way interactions was significant. Again P300 peak latencies were significantly shorter for other (412 ms) compared to self (444 ms) stimulus (*F*_1,10_ = 6.61, *p* = 0.028). No main effect of *priming* (*F*_1,10_ = 0.00, *p* = 0.972), but a significant *priming* × *stimulus* interaction (*F*_1,10_ = 7.82, *p* = 0.019) was present. The latter reflected larger latency differences between self (460 ms) and other (395 ms) after LKM compared to ORT (427 vs. 429 ms). Priming effects on P300 do not support hypothesis 3 that differences between self- and other-related P300 would be reduced by a short LKM session as compared to a control procedure.

### Correlation Analysis

To test for associations between self–other integration and self-reported compassion (hypothesis 4) and between self–other integration and meditation practice (hypothesis 5), as well as to assess potentially confounding factors, we correlated these measures with P300 differences (self-related P300 minus other-related P300) at Pz as well as the pooled region of interest defined above (**Table 3**).

**Table 3:** Correlation analysis for P300 differences.

**Scale**	**P300 Self–P300 Other**			
	
	**Pz**		**Pool**	
		
	***r***	***p***	***r***	***p***
CLS-H^a^	-0.52	**0.006**	-0.36	0.051
CLS-O^a^	0.01	0.483	-0.04	0.422
SCS^a^	-0.44	**0.023**	-0.40	**0.037**
IOS^a^	-0.13	0.277	-0.16	0.234
Med. Exp.^b^	-0.70	**0.012**	-0.76	**0.005**
LKM Exp.^b^	-0.58	**0.040**	-0.57	**0.041**


*Pool, average of Pz, POz, P1, P2, PO3, PO4 electrodes; CLS-H, Compassionate Love Scale, stranger-humanity version; CLS-O, Compassionate Love Scale, specific close other version; SCS, Self-Compassion Scale; IOS, Inclusion of Other in Self Scale; Med. Exp., meditation experience in months; LKM Exp., LKM experience in months; *p*-values (one-tailed) ≤ 0.05 are highlighted in bold face type. ^a^*r* = Spearman’s correlations (*n* = 22). ^b^Partial correlations controlling for age (*n* = 11).*

**FIGURE 6:**
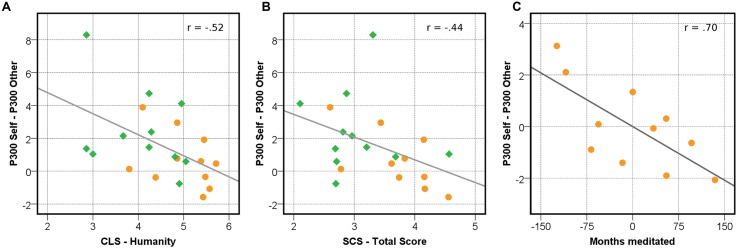
**Correlations between P300 differences, self-report measures, and meditation experience.** **(A)** Correlation between compassionate love for humanity and differences in P300 amplitudes (self–other). **(B)** Correlation between self-compassion and P300 self minus P300 other. **(C)** Partial correlation between P300 differences and months of general meditation practice, controlled for age. In all figures, P300 amplitudes at Pz are displayed. CLS, Compassionate Love Scale; SCS, Self-Compassion Scale.

Self-compassion and compassionate love for all of humanity yielded significant and large negative correlations with P300 differences at Pz (**Figure 6**). Compassionate love for close other and inclusion of other in self were not correlated significantly with P300 differences. This pattern of results remained when removing an outlier with high P300 differences.

Furthermore, we investigated whether P300 effects in meditators were related to the extent of individual meditation practice, measured in months of general meditation practice and months of LKM practice. Since meditation experience increases with age, we computed partial correlations with age as control variable (**Table 3**). Correlations were large and significant for P300 differences, indicating that participants with longer meditation experience exhibited smaller differences between P300 elicited by self and other (**Figure 6**). These associations were slightly stronger for general meditation practice than for LKM.

The following potentially confounding variables did not correlate significantly—and only to a small extent—with P300 elicited by close other: years of acquaintanceship, recency of last meeting, rated similarity between self and other, and rated pleasantness of close other image (*r*_s_ ≤ 0.12 and ≥-0.22, *p* ≥ 0.28). A small to medium, though not significant, negative relationship was observed between frequency of meeting and P300 mean amplitudes at Pz (*r*_s_ = -0.36, *p* = 0.099), which was small for pooled amplitudes (*r*_s_ = -0.19, *p* = 0.396). Correlations of P300 mean amplitudes elicited by self-stimulus with rated pleasantness of self-image were small and not significant (Pz: *r*_s_ = -0.01, *p* = 0.976; pool: *r*_s_ = 0.07, *p* = 0.748).

These correlation results support the predictions that smaller P300 differences would be related to more extensive meditative practice (hypothesis 5) and to higher levels of self-reported compassion toward oneself and others (hypothesis 4).

## Discussion

According to current models in social neuroscience, prosocial human qualities such as empathy and compassion are based on shared representations of self and other (Preston and Hofelich, 2012). Furthermore, previous studies have shown that compassionate responses depend on the closeness of the other, e.g., whether she/he belongs to an in-group (Hein et al., 2010), or is personally close (Hein et al., 2010; Meyer et al., 2012). Based on this research, we hypothesized that the cultivation of prosocial affect through LKM involves a rebalance of self- and other-related processing by increasing the overlap or integration of representations of self and other (Decety and Sommerville, 2003). This is consistent with the phenomenology of LKM, which involves extending a feeling of love and kindness from the self toward close and even unfamiliar others (Salzberg, 1995; Wallace, 2001). In order to investigate whether a rebalance in self- and other-related processing is associated to the experience and cultivation of prosocial affect, we recorded ERPs elicited by the self-face and a close other’s face, which usually yield a preferential processing of the self in the P300. The sample of 12 long-term practitioners of LKM and 12 matched controls additionally completed subjective measures of self-compassion and compassionate love. We hypothesized that, compared to controls, meditators would experience more compassion toward the self and others in general, accompanied by a rebalance of self- and other-related processing (i.e., smaller differences between self- and other-related P300), while a short LKM state compared to a control state would additionally increase self–other integration. Furthermore, we tested whether smaller P300 differences (self–other) would be correlated with extent of meditative practice in meditators as well as with self-reported compassion for oneself and others across the entire sample.

Regarding self-report data, meditators reported more compassion for the self and others in general, while there were no significant differences in closeness to and compassion for the specific close other. This is consistent with the aim of LKM to cultivate kindness and compassion in an unbiased way (Salzberg, 1995). Regarding the ERP data, we found that a self-related stimulus—the self face—elicited larger P300 amplitudes, even when compared to the face of a close other. This extends previous studies where stimuli in the control condition represented familiar, not personally known others (e.g., Tacikowski and Nowicka, 2010). We then tested whether the size of this effect would be related to the cultivation and experience of prosocial affective qualities. First, we compared ERPs in LKM practitioners and matched controls. While there was no general reduction in P300 differences between self and other in meditators, a significant interaction of group, stimulus, and electrode location indicated that these differences were smaller in meditators at posterior, but not frontal midline locations (**Figure 3**). This effect was mainly driven by a differing topography of the P300 component in the self condition, which had a frontocentral peak in meditators and a more dispersed central to centroparietal focus in controls (**Figure 2**). This pattern suggests that only a sub-process of self- vs. other-related processing with a specific topography differed between both groups. It is a well-supported finding that several subcomponents contribute to the P300 (Polich, 2007), with a wide array of neural generators (Linden, 2005). Due to the posterior distribution of group differences one could speculate that a process related to the P3b (Polich, 2007) was preferentially recruited for the self stimulus in controls, and less so in meditators. As the P3b is typically elicited by target stimuli, this would imply that the self-face had a target like effect in controls, whereas it had a distracter effect—stronger but similar to the other-face—in meditators.

Since the simple interaction effects analysis showed strongest group effects (critical significance) at posterior electrode locations, we ran additional correlation analyses with amplitude values from this location to further characterize relations between this effect and the cultivation and experience of prosocial affect. Within meditators we observed a strong negative correlation between P300 differences and the extent of individual meditation practice (**Figure 6**). Taken together, these findings provide support that a change in sub-components of self vs. other-related processing is associated to prolonged meditative practice.

As the P300 presumably reflects attentional resource allocation and meditation training has been associated with changes in attention (Brefczynski-Lewis et al., 2007; Tang et al., 2007)—which has also been indexed in a P300 paradigm (Cahn and Polich, 2009)—one might argue that these results reflect general changes of attentional mechanisms. However, no significant group effects were found for the oddball stimuli in general (i.e., target, distracter, standard, see Supplementary Material). Instead, the only group effect emerged within the category of distracter stimuli as a spatially dependent reduction of differences between self vs. other-related P300 in meditators. Thus, this effect seems to be specific to the self-relatedness of the stimulus.

Furthermore, the notion that P300 differences between self and other stimuli might indeed reflect the representational closeness or overlap of self and others representations was supported by correlation results: across groups P300 differences were negatively correlated with compassionate love for others and with self-compassion (**Figure 6**). Both of these concepts involve a more unbiased way of relating to the self and others: compassionate love refers to an attitude of kindness and concern that does not depend on the specific relationship with others (Sprecher and Fehr, 2005), and self-compassion involves a de-emphasis of the individual self in favor of interdependent and shared aspects of identity (Neff, 2008).

These results are cross-sectional in nature and do not warrant strong conclusions about causality. In order to directly investigate causal effects of LKM on self- and other-related processing, we compared a short loving-kindness state to a non-affective control priming (ORT). These state inductions were done directly before assessment of ERPs, and we assumed that the LKM state would reduce differences between self- and other-related P300. The effects of these priming conditions were investigated only in meditators based on the assumption that the state induction would be stronger and thus effects on the ERP measure more likely in trained practitioners of LKM. However, no significant difference between LKM and ORT in P300 amplitudes was observed. Several explanations might account for this: first, as both conditions were administered in a within-subjects design, carryover effects might have occurred. Second, the LKM state effects might have been too short-lived (or suppressed by the subsequent task) in order to be captured in our paradigm. Third, the ORT priming might have elicited processes which also increase self–other overlap, such as perspective taking (Davis et al., 1996). Fourth, high trait levels of self–other integration in meditators (as indicated by the baseline comparison with controls) might have reduced the potential for state changes (e.g., due to ceiling effects); future studies should thus investigate priming effects also in novice practitioners. Finally, it is possible that LKM does not have a direct effect on self- and other-related processing and that our findings in the group comparison and correlation analysis have a different origin.

An important limitation of the present study is the relatively small sample size, which might result in undetected effects or contribute to inflated effect sizes (Button et al., 2013). However, previous research on long-term outcomes of meditation has relied on similar sample sizes (Lutz et al., 2004; Nielsen and Kaszniak, 2006), probably due to the difficulty of recruiting larger samples with more extensive meditation training. Thus, studies replicating and extending the present results, ideally in a longitudinal study design, are needed to confirm this first association between the practice of meditation and increased self–other integration.

All meditators in our sample reported to complement LKM with other practices such as mindfulness meditation. Since mindfulness meditation has been found to attenuate self-referential processing (e.g., Farb et al., 2007), it is possible that at least part of the effects in the group comparison are due to this practice. This would be consistent with contemplative accounts, which suggest that LKM and mindfulness meditation mutually support each other (Wallace, 2001; Salzberg, 2011). Thus, future studies should try to disentangle specific and common effects of these practices on self- and other-related processing.

In spite of these limitations, the current study provides some evidence that a reduced focus on the individual self and stronger self–other integration might be an underlying mechanism of prolonged meditative practice. Such a mechanism would have important implications. First, it might at least partially explain the effects of meditation on mental health and well-being. Excessive self-focus and feelings of isolation are a hallmark of mental disorders such as depression, and practices such as mindfulness meditation and LKM are increasingly being regarded as an effective treatment of these conditions (Papageorgiou and Wells, 2004; Galante et al., 2014; Goyal et al., 2014). Moreover, it has been argued that a focus on the individual self is the source of fluctuating happiness, whereas a more integrated and connected perception of the self gives rise to more durable happiness (Dambrun and Ricard, 2011). Second, such a mechanism might explain some of the effects of meditation in the social domain, including effects on relationship quality, empathy, compassion, and altruistic behavior (Block-Lerner et al., 2007; Mascaro et al., 2015). However, is has also been argued that self–other distinction is crucial for effective prosocial action as well as to avoid self-related empathic distress, and on the long run, “empathy fatigue” (Klimecki and Singer, 2011; Singer and Klimecki, 2014). Thus, future studies should address how these processes of self–other distinction and integration relate to each other.

In summary, the results support the notion that prolonged meditative practice is associated to a rebalance of sub-components of self–other-related processing. While effects of meditation on self-related processing have been found previously (Farb et al., 2007; Berkovich-Ohana et al., 2012; Dor-Ziderman et al., 2013), our study provides a potential answer to the intriguing question of how the solitary practice of meditation may increase social human qualities such as empathy and compassion (Kristeller and Johnson, 2005). In particular, the results are consistent with the notion that habitually de-emphasizing distinctions between self and others through LKM may increase compassionate connectedness with others. While a strong correlation with the amount of meditative practice is supportive of this interpretation, it is not possible to directly infer a causal role of LKM from these cross-sectional results. Furthermore, the interpretation is limited by a relatively small sample size and the fact that the specific effects of LKM cannot be separated from other reported practices. Nevertheless, our study provides first evidence for a relation between meditative practice, self–other integration, and the experience of prosocial affect. We hope that future studies, in particular studies with longitudinal designs, will further elucidate these links, as it seems a promising endeavor to investigate the nature of meditation effects in the self–other domain.

## Author Contributions

JN developed the initial concept and JN and SS acquired the funding for the study. F-MT, JN, and SS developed details of the design and methods. F-MT and JN implemented the study, acquired and analyzed the data. F-MT drafted the manuscript, and all authors contributed to revising it.

## Conflict of Interest Statement

The authors declare that the research was conducted in the absence of any commercial or financial relationships that could be construed as a potential conflict of interest.
