# Supplementary material for: Decentering the Self? Reduced Bias in Self- vs. Other-Related Processing in Long-Term Practitioners of Loving-Kindness Meditation
Source: Front Psychol. 2016 Nov 21;7:1785. doi: 10.3389/fpsyg.2016.01785 (PMC5116565; doi:10.3389/fpsyg.2016.01785)
Supplement: Supplementary file 1 [file Data_Sheet_1.docx]

Supplementary Material

Decentering the self? Reduced bias in self- versus other-related processing in long-term practitioners of loving-kindness meditation

Fynn-Mathis Trautwein*, José Raúl Naranjo, Stefan Schmidt

*** Correspondence:** Fynn-Mathis Trautwein: trautwein@cbs.mpg.de

# Supplementary Figures and Tables

## Supplementary Figures


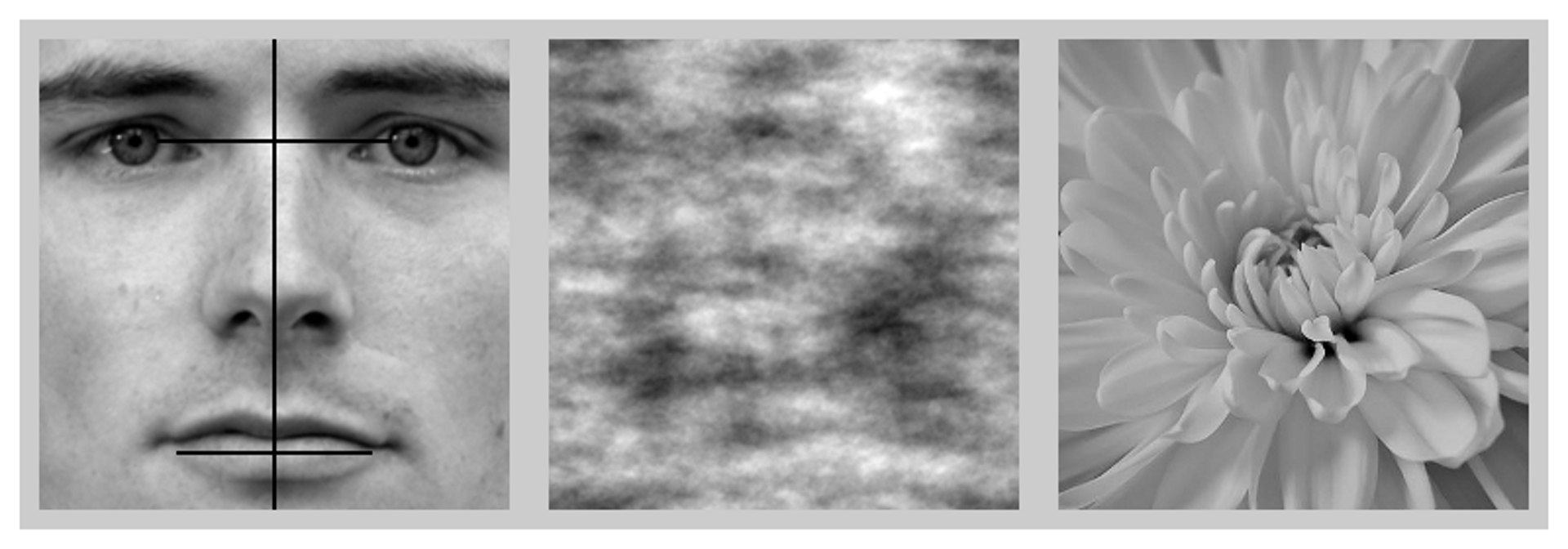


**Supplementary Figure 1.** Example stimuli for each stimulus category of the oddball paradigm. Left: distracter face with standard scaling mask used to define the pupil to mouth distance and the midline of the face. Middle: scrambled face (standard stimulus). Right: flower (target stimulus).

**
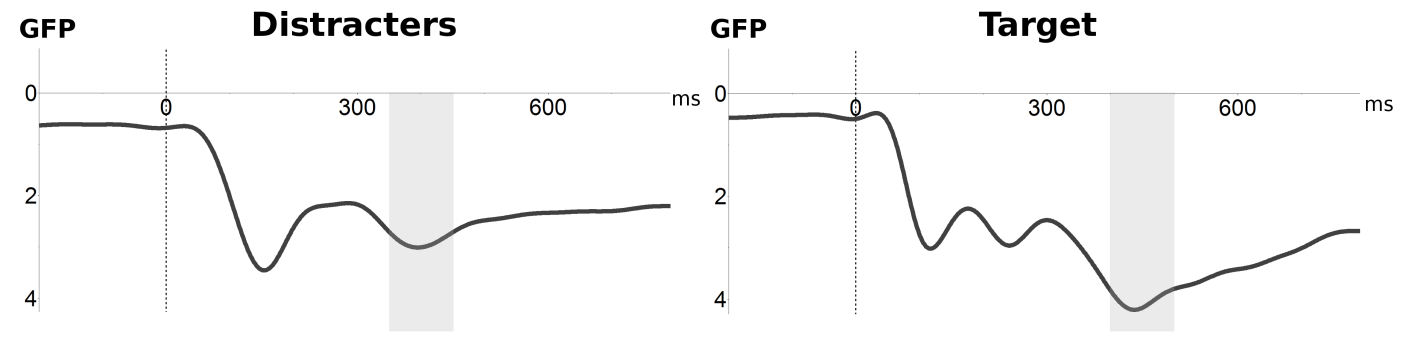
 Supplementary Figure 2.** Global field power for the grand average of ERPs elicited by both distracter (self and other faces) and target stimuli in both groups in the no priming condition. The intervals chosen for mean amplitude measurements (350 ms to 450 ms for distracters; 400 ms to 500 ms for targets and standards) are marked by the gray area.


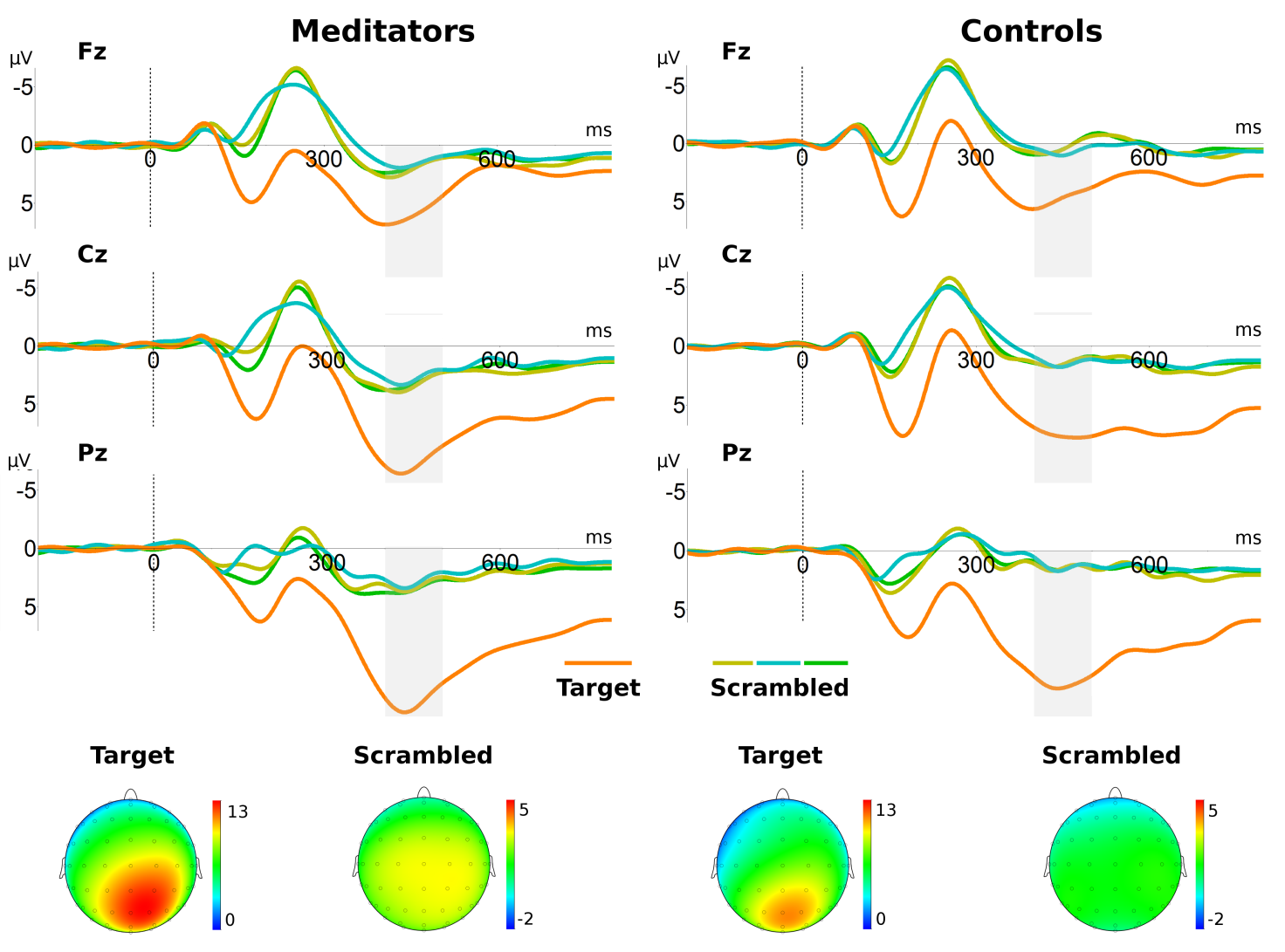
**Supplementary Figure 3.** ERPs elicited by target and scrambled stimuli. The target stimulus (flower) elicited a P300 component with a posterior scalp distribution. For scrambled stimuli, no P300 component was present. For topographic maps of scrambled stimuli, these were averaged, as statistical and descriptive analysis did not yield any differences between them.


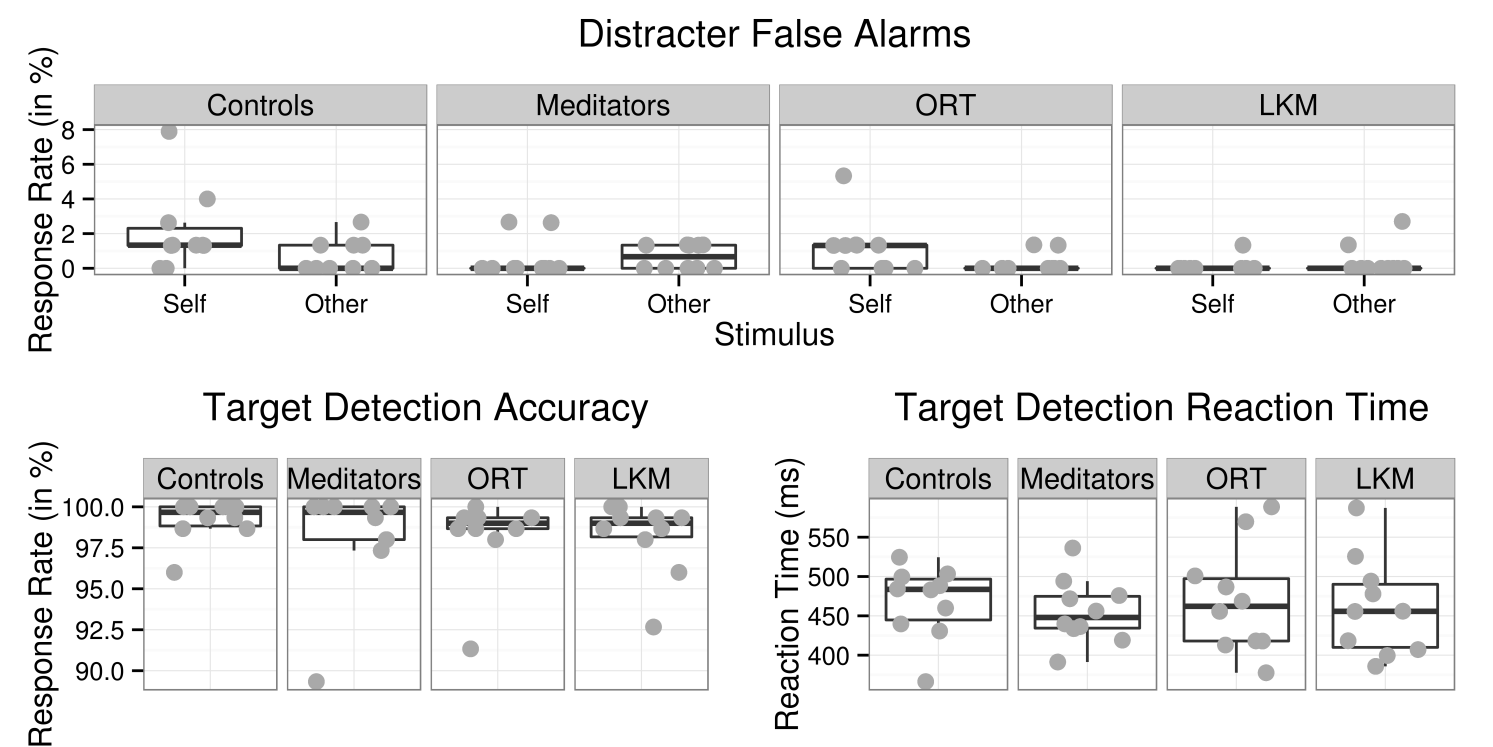


**Supplementary Figure 4.** Behavioral data. Upper panel: Frequency of responses (“false alarms”) to self- and other-face stimuli for meditators and controls in the baseline (no priming) measurements in both groups, as well as for meditators after the two priming conditions (LKM = Loving-Kindness Meditation, ORT = Other Referential Thinking). Lower panel: Accuracy (left) and reaction time (right) for target detection in the no priming and priming assessments.

## Supplementary Tables

**Supplementary Table 1. ANOVA of P300 mean amplitudes of oddball stimuli.**

| Factor | df | F | p | η^2^ |
| --- | --- | --- | --- | --- |
| Group | 1 | .53 | .475 |  |
| Stimulus | 2 | 38.47 | **.000** | .87 |
| Electrode | 1.62 | 25.89 | **.000** | .56 |
| G x S | 2 | 1.82 | .182 | .08 |
| G x E | 1.62 | .76 | .451 | .04 |
| S x E | 2.54 | 24.29 | **.000** | .55 |
| G x S x E | 2.54 | .30 | .794 | .02 |
| p-values ≤ .05 are highlighted in bold face type. | | | | |
